# Cognitive Reappraisal is More Effective for Regulating Emotions than Moods

**DOI:** 10.1007/s42761-025-00310-3

**Published:** 2025-06-06

**Authors:** Samuel Meyers, Danfei Hu, Maya Tamir

**Affiliations:** The Hebrew University of Jerusalem, Mount Scopus, 9190501 Jerusalem, Israel

**Keywords:** Cognitive reappraisal, Emotion regulation, Mood regulation, Intentionality

## Abstract

Cognitive reappraisal is one of the most commonly used and effective emotion regulation strategies. This is partly because reappraisal targets a key characteristic of emotion – namely, what an emotion is about (i.e., intentionality). It is commonly assumed that whereas emotions have intentionality because they are about something, mood states do not have intentionality because they are typically not about anything in particular. Given that reappraisal targets intentionality, and mood states theoretically lack intentionality, we hypothesized that reappraisal would be less effective in regulating moods than in regulating emotions. We tested this hypothesis in two studies, one in which we assessed naturally occurring experiences and regulation in daily life (Study 1, *N* = 184), and another in which we manipulated experiences and regulation in a controlled experiment (Study 2, *N* = 65). In both studies, we compared the effects of cognitive reappraisal with those of distraction, which is another effective emotion regulation strategy that does not target intentionality. As expected, cognitive reappraisal, but not distraction, was more effective in regulating emotions than moods. These findings suggest that the regulation of emotions may differ from the regulation of moods and that strategies that may be effective in regulating one type of affective state may be less effective in regulating another.

**Supplementary Information:**

The online version contains supplementary material available at 10.1007/s42761-025-00310-3.

One of the most commonly used and effective strategies to influence emotions is cognitive reappraisal (Ford & Troy, 2019; Webb et al., 2012). It involves altering the meaning assigned to an emotional event, so that its emotional impact changes (Gross, 1998a). Cognitive reappraisal is effective, in part, because it targets an essential component of emotions – namely, what they are about. Emotions are considered intentional states, which means that they are about something, in particular (Frijda, 1994). Moods, on the other hand, are considered more diffuse and unintentional (Clore et al., 1994). If cognitive reappraisal targets what the affective state is about, and emotions, unlike moods, are about something, cognitive reappraisal may not be equally effective in regulating moods as it is in regulating emotions. This hypothesis was the focus of the current investigation.

## Cognitive Reappraisal in Emotion Regulation

Emotions result from the appraisals of what they are about (e.g., Yeo & Ong, 2024). According to the process model of emotion regulation (Gross, 1998b), one way in which emotions can be regulated, known as cognitive reappraisal, is by changing the meaning assigned to them. For instance, if someone is irritated about being stuck in traffic, they can reappraise the situation as an opportunity to enjoy music on the radio, which would decrease irritation. Cognitive reappraisal is particularly effective in regulating emotions – in the laboratory (e.g., Nasso et al., 2022; Uusberg et al., 2023) and in daily life (e.g., Boemo et al., 2022) – because it targets the very thing emotions are about.

## Cognitive Reappraisal in Mood Regulation

Cognitive reappraisal has also been applied broadly as an affect regulation strategy (Gross et al., 2019; Troy et al., 2023). Affect is an umbrella term encompassing various states, such as emotions and moods (Scherer, 1984). As affective states, emotions and moods both involve subjective experiences that vary in valence (i.e., degree of pleasantness or unpleasantness) and arousal (i.e., degree of activation), reflecting evaluations of what is happening (Russell, 1980).

Although emotions and moods share many features, they are often considered distinct in some respects (for a review, see Meyers & Tamir, 2024). Whereas some have proposed that emotions and moods differ in duration (Ekman, 1994) or intensity (Panksepp, 1994), there is a general consensus that intentionality is a major characteristic that distinguishes emotions from moods (Frijda, 1994; Russell, 2003; Siemer, 2009; Watson, 2000). Emotions are considered high in intentionality (i.e., they are typically about something; Frijda, 1994). For instance, someone stuck in traffic might be irritated about the traffic, in particular. In contrast, moods are considered low in intentionality (i.e., they are not about anything in particular). For instance, a person may wake up feeling irritated, without being irritated about anything in particular. Intentionality has been challenged the least in the literature as a factor that distinguishes emotion from mood (see Meyers & Tamir, 2024). For instance, Parkinson et al., (1996, p. 8) referred to intentionality as the “underlying distinction between emotion and mood”. Siemer (2005, p. 816) described intentionality as “the core differentiating feature” between emotions and moods with a “widely shared consensus”.^1^

In many accounts of mood regulation, cognitive reappraisal (sometimes referred to as positive reframing) has been identified as an effective strategy (e.g., Larsen, 2000; Morris & Reily, 1987; Parkinson & Totterdell, 1999; Thayer et al., 1994). However, if moods, unlike emotions, are low in intentionality, cognitive reappraisal – which directly targets intentionality – should theoretically be less effective in regulating moods than emotions. Consistent with this hypothesis, Rottenberg and Gross (2007) posited that emotion regulation should relate to an object more than mood regulation. Manstead and Fischer (2000) argued that unlike emotions, reappraisal may not effectively regulate moods, precisely because they lack intentionality. The current investigation builds on these ideas, testing them empirically, for the first time.

Testing whether reappraisal is equally effective in regulating emotions and moods is important. First, moods characterize much of our affective lives (Davidson, 1994). Also, attempts to repair bad moods are frequent (Tice & Bratslavsky, 2000), and so identifying what leads to effective mood regulation is important. Second, mood regulation is particularly relevant in psychopathology (Joormann & Siemer, 2014). Mood disorders involve maladaptive mood states that require effective regulation (Joorman & Gotlib, 2010). If reappraisal is less effective in regulating mood states (vs. emotions), this could carry clinical implications.

## The Current Investigation

We hypothesized that cognitive reappraisal would be more effective in regulating emotions than moods. We tested this hypothesis by focusing on the regulation of sadness, because unlike other discrete emotions (e.g., shame), people can experience sadness as an emotion or as a mood state (Siemer, 2005) Also, sadness has often been studied and manipulated both as an emotion (e.g., Millgram et al., 2015) and as a mood (e.g., Joorman & Siemer, 2004). Sadness is also a common experience in daily life (Oatley & Duncan, 1994), and in mental disorders (e.g., depression).

We tested our hypothesis in two studies. Study 1 tested effects of cognitive reappraisal on regulating naturally occurring emotions and moods in daily life. Study 2 tested the causal effect of cognitive reappraisal in an experiment, with manipulated emotions and moods. To distinguish between emotions and moods, we assessed or manipulated intentionality, with higher intentionality indicating emotions and lower intentionality indicating moods.

In both studies, to test whether effects were specific to cognitive reappraisal, we compared it to distraction, which involves diverting attention away from the event or the experience. Distraction is another effective emotion regulation strategy (Webb et al., 2012), but one that does not target what the affective state is about (Gross, 1998b), therefore serving as a reasonable comparison strategy to reappraisal. We expected cognitive reappraisal, but not distraction, to be more effective in regulating emotions than moods.

## Study 1

In an ecological momentary assessment (EMA) study, we tested whether the use of cognitive reappraisal in everyday emotion regulation was associated with a subsequent differential decrease in sadness, depending on whether sadness was an emotion or a mood (i.e., the experience had or did not have intentionality).

### Method

Study 1 was conducted as part of a larger project examining daily emotion regulation (e.g., Hu et al., 2025, Study 2). We describe parts of the project that are relevant to this research. This study was approved by the ethics committee of the Faculty of Social Sciences at The Hebrew University of Jerusalem and our hypothesis, methods, and analysis plan was preregistered at https://aspredicted.org/w6zw-j9ft.pdf.^2^

#### Participants

The sample included 184 students (*M*_age_ = 24.28; *SD*_age_ = 2.37; 81.52% female; 54.89% currently employed; 86.41% single; 51.09% diagnosed with current Major Depressive Disorder). Participants were recruited through the university’s experimental registration system. Sample size was determined by the key question of the larger project. A summary-statistics-based power analysis (Murayama et al., 2022) indicated that a minimum sample of 120 participants is necessary to detect an effect for the larger research question (*b* = −0.06; *t* = −2.44; random intercept only model), with a power of 0.80. We oversampled to account for possible dropouts and exclusions.

#### Procedure

The project included a baseline assessment, a 10-day EMA portion, and a follow-up assessment. Here, we focus specifically on the EMA portion. Using the SEMA3 application (Koval et al., 2019), participants were notified to complete EMA surveys on their smartphones. These notifications occurred six times daily over a span of 10 days, from 10 am to 10 pm. We used a stratified random interval approach to ensure an even distribution of surveys throughout the day, dividing each day into six equal time windows. Within each window, participants received a prompt at a randomly selected time within the first 45 min. To prevent consecutive surveys from being too close together, we programmed a 30-min interval between prompts. Once prompted, participants had 30 min to complete the survey. Participants received up to 50.5 USD or 19 study credits for completing the entire 10-day EMA study.

From 184 participants, we received a total of 8,275 EMA surveys. Following our preregistration, we excluded individual surveys in which participants failed an attention check, had zero variance in responses, or were defined as careless responses (submitted in less than one minute). Therefore, 66 responses (0.80%) were excluded, resulting in 8,206 EMA surveys (compliance:* M* = 74.33%, *SD* = 23.14%).

#### Measures

The EMA measures targeted experiences in the last two hours and were rated on a scale of 1 (= *not at all)* to 9 (= *very much)*.

##### Experienced Sadness

Participants rated their experienced sadness by responding to the following item, “In the last two hours, to what extent did you feel sadness?”

##### Affect Type (Emotion vs. Mood)

Participants indicated whether they felt sad about something in particular (yes/no). We considered an affirmative response as reflecting sadness as an emotion and a negative response as reflecting sad mood.

##### Cognitive Reappraisal and Distraction

Participants indicated the extent to which they implemented specific strategies to decrease their unpleasant feelings, including reappraisal (i.e., “I changed the way I thought about things, so they became less negative”) and distraction (i.e., “I tried to distract myself from what was making me feel bad and think about something else”).

#### Analytic Approach

To account for the hierarchical structure of the data (i.e., EMA surveys nested within persons), we conducted multilevel modeling analyses in the R statistical programming software (R Core Team, 2013), using the lmerTest (Kuznetsova et al., 2017) package. Confidence intervals were obtained using the sjPlot (Lüdecke, 2023) package. Missing data were accounted for by using full information restricted maximum likelihood estimation (e.g., Raudenbush & Bryk, 2002). Effect sizes were indicated by semi-partial *R*^2^, using the r2 glmm package (Jaeger et al., 2017). Significant interactions were further probed by assessing the simple effects, using the reghelper package in R (Hughes, 2017). In all models, all Level 1 variables were person-mean centered. As pre-registered, each model included random slopes for continuous Level-1 predictors. When the models did not converge, we removed the random effect explaining the least variance (i.e., distraction intensity in the second model; Barr et al., 2013). Below, we describe the details of each model and the respective findings.

During data collection, the events of October 7^th^ occurred, and a subsequent war broke out that lasted throughout data collection. Given that the onset of war is likely to influence emotional reactions, which were the target of our investigation, we included a binary variable capturing whether data was collected before or after October 7^th^ as a potential moderator. As these events could not be anticipated, this change in our analysis plan could not be pre-registered.

## Results

### Did Affect Type Moderate the Effectiveness of Reappraisal?

To test whether the effectiveness of reappraisal in reducing sadness varied by whether the sadness was an emotion or a mood, we adopted an autoregressive approach (Brose et al., 2015; Koval et al., 2023; Kuppens et al., 2012), where we entered reappraisal intensity at time *t*−1, affect type at time *t*−1, war onset, and their interactions as predictors, and experienced sadness at time *t* as the outcome, while controlling for experienced sadness at time *t*−1.^3^ Given that our sample included clinically depressed individuals, we also controlled for participants’ depressive status. The pattern of results was consistent whether we controlled for depression or not.

**Fig. 1:**
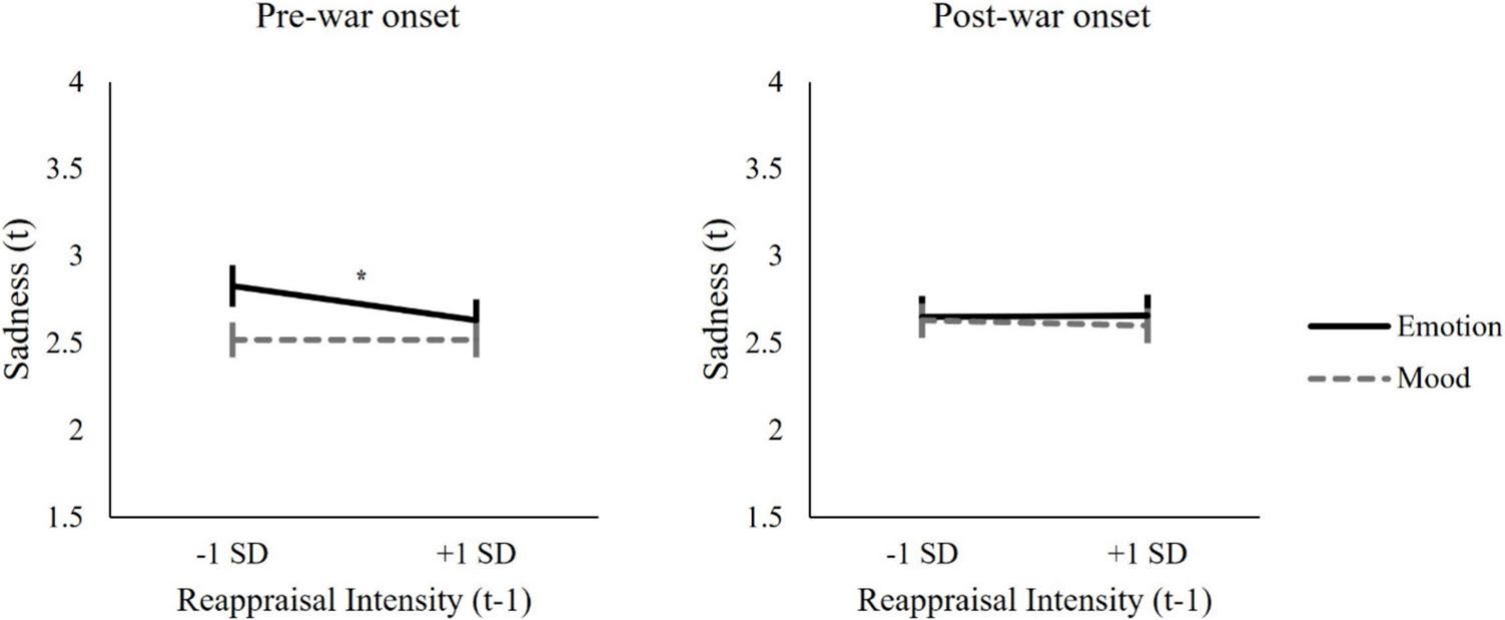
Prospective Associations Between Cognitive Reappraisal and Sadness in Daily Life, when Sadness was Experienced as an Emotion vs. Mood. *Note.* Error bars represent standard error. * indicates *p* < 0.05

Given our hypothesis, our discussion of the results focuses on the interaction and comparison of simple effects. All effects are reported in Table 1. We found a three-way interaction between reappraisal intensity, affect type, and war onset, *B* = 0.03, *SE* = 0.01, *p* = .028, 95% CI [0.00, 0.06]. As shown in Fig. 1, simple slope tests indicated that, as we predicted, before the war, greater use of reappraisal was prospectively associated with greater decreases in the emotion of sadness at *t*, *B* = −0.10, *SE* = 0.04, *p* = .003, 95% CI [−0.18, −0.03]. However, there was no significant association between reappraisal intensity and changes in sad mood, *B* = 0.00, *SE* = 0.03, *p* = .897, 95% CI [−0.05, 0.05]. This pattern, however, changed after the onset of war, such that reappraisal intensity was no longer associated with decreases in sadness either as an emotion, *B* = 0.01, *SE* = 0.03, *p* = .817, 95% CI [−0.06, 0.07], or as a mood, *B* = −0.01, *SE* = 0.03, *p* = .586, 95% CI [−0.07, 0.03]. These results suggest that before the onset of war, reappraisal was more effective in regulating sadness as an emotion than mood.

**Table 1:** Results of Model Predicting Prospective Changes in Sadness from Reappraisal, Affect Type, and War Onset

*Predictors*	*Estimates*	*std. Error*	*CI*	*p*	*R*^*2*^
(Intercept)	2.63	0.07	2.49–2.78	< .001	—
Reappraisal intensity (t-1)	−0.03	0.02	−0.06 – 0.01	.102	0.001
Affect type (t-1)	0.06	0.03	0.00–0.12	.035	0.001
War onset	0.00	0.07	−0.14 – 0.15	.957	0.000
Sadness (*t*−1)	0.25	0.02	0.20–0.29	< .001	0.048
Depressive status	0.82	0.07	0.68–0.97	< .001	0.231
Reappraisal intensity (t-1) × Affect type (t-1)	−0.02	0.01	−0.05 – 0.01	.145	0.000
Reappraisal intensity (t-1) × War onset	0.02	0.02	−0.01 – 0.06	.149	0.001
Affect type (t-1) × War onset	−0.04	0.03	−0.10 – 0.01	.123	0.001
**Reappraisal intensity (t-1)** ** × Affect type (t-1)** ** × War onset**	**0.03**	**0.01**	**0.00–0.06**	**.028**	**0.001**

Affect Type was effect coded (“mood” = −1; “emotion” = 1), depressive status was effect coded (“Control” = −1; “Depression” = 1), and War onset was effect coded (pre-onset of war = −1; post-onset of war = 1). Effect of interest (the three-way interaction) is bolded

#### Tests of Alternative Explanations

To test the robustness of our findings, we examined alternative explanations. First, to test whether the effect of affect type was specific to reappraisal, we repeated the analysis above, with distraction as the predictor. As expected, there was no significant interaction between distraction intensity, affect type, and war onset, *B* = −0.00, *SE* = 0.01, *p* = .907, 95% CI [−0.02, 0.02], indicating that the effects of distraction were similar when people regulated emotions versus moods before and after the war (see the online supplemental materials for the full results).

In our data, experiences of sadness as a mood were less intense than experiences of sadness as an emotion, which is consistent with predictions of some existing theories (Panksepp, 1994). Given that the efficacy of reappraisal can vary by intensity (Shafir et al., 2015), we proceeded to test whether the effect of affect type in our data could be explained by differences in emotional intensity. We conducted a similar analysis as above, using reappraisal intensity at time *t*−1, experienced sadness at time *t*−1, war onset, and their interactions to predict experienced sadness at time *t*. There was no significant three-way interaction, *B* = 0.01, *SE* = 0.01, 95% CI [−0.00, 0.03], *p* = .111. Therefore, regardless of war onset, differences in emotional intensity did not explain the differential efficacy of reappraisal in regulating emotions versus moods (see the online supplemental materials for the full results).

## Discussion

The findings of Study 1 suggest that cognitive reappraisal may be more effective for regulating sadness as an emotion than as a mood in daily life. Nonetheless, Study 1 had several limitations. First, the design does not allow us to draw causal claims. Second, distraction was not effective in regulating emotions or moods in our data. Third, although emotional intensity did not drive the effect of affect type, emotions and moods in daily life may differ in ways other than intentionality per se. Fourth, we do not know why the effect did not persist during the war. It may be that participants used reappraisal to decrease unpleasant emotions other than sadness (e.g., anxiety). This is likely given that people may be motivated to experience sadness, following national loss, in order to feel connected with their ingroup (Porat et al., 2016). This possibility that people may have used reappraisal to decrease emotions other than sadness is consistent with the finding that using reappraisal after the war was unrelated to changes in sadness. Another possibility is that people turned to social reappraisal (e.g., Sahi et al., 2025) after the war.^4^ Study 2 addresses many of the limitations of Study 1.

## Study 2

In Study 2, we tested the causal effects of cognitive reappraisal and affect type (i.e., emotions vs. moods) in an experimental design. First, we manipulated intentionality to induce sad emotions or sad moods. To do so, we used instrumental and lyrical music clips, which were similarly intense. Sad instrumental music can elicit a sad mood (rather than an emotion) because it lacks semantic content, and therefore, is not about anything in particular (Manstead & Fischer, 2000). In contrast, sad lyrical music includes sad semantic content and points to something the listener can feel sad about. We expected the sad lyrical music to be higher in intentionality, and therefore, to more likely reflect emotion, compared to sad instrumental music, which we expected to reflect mood. Utilizing sad instrumental and lyrical music allowed us to control for the source and intensity of feelings, isolating differences in intentionality.

Second, we trained participants to implement reappraisal or distraction, and manipulated strategy use in different trials. This ensured that participants knew how to use each strategy and tested the causal effect of doing so. Third, as there is some debate over the specificity or discreteness of mood states (Panksepp, 1994; Watson & Clark, 1994), we used both discrete items and indices of valence and arousal to measure affective experiences.

### Method

Study 2 employed a 2(affect type: emotion vs. mood) × 2(strategy: reappraisal vs. distraction) within-subject experimental design. This study was approved by the ethics committee of the Faculty of Social Sciences at The Hebrew University of Jerusalem and our hypothesis, methods, and analysis plan were preregistered at https://aspredicted.org/8ztp-j964.pdf.

#### Participants

The sample included 65 participants (*M*_age_ = 24.38, *SD*_age_ = 2.91; 83.07% female; 49.23% single). Participants were undergraduate students, recruited through the university’s experimental registration system, who did not have any attention or hearing deficits. A power analysis indicated that a sample of 64 would be required to detect a small effect size (*f* = 0.15), with an alpha of 0.05, and power of 80%. We preregistered to collect a minimum of 64 participants. We oversampled and recruited 88 participants through the university experimental registration system. Participants received three psychology study credits or 8.00 USD for their participation. As the study was conducted online, we inserted a total of four attention (to select a specific multiple-choice answer) and seven manipulation checks (detailed below) to ensure participants’ engagement and the validity of the data. Seven participants failed at least one attention check, and 16 participants failed more than half of the seven manipulation checks. As pre-registered, these participants were excluded, resulting in a sample of 65 participants.

#### Measures

Unless indicated otherwise, continuous measures were rated on a scale of 1 (= *not at all)* to 9 (= *very much)*.

##### Current feelings

To assess baseline sadness, participants rated the extent to which they currently felt sad, gloomy, and downhearted (α = 0.90; Millgram et al., 2015). As filler items, participants also rated the extent to which they currently felt happy, joyful, and cheerful.

##### Affective reactions to music clips

After each clip, participants rated how sad and gloomy they felt while listening to each clip (α_pre-regulation_ = .96; α_regulation_ = .97). We also assessed valence (*1* = *very negative, 9* = *very positive*) and arousal (*1* = *low energy, 9* = *high energy*). As filler items, participants also rated the extent to which they experienced happiness and cheerfulness.

##### Music clips

Sixteen music clips, eight instrumental and eight lyrical, were used to elicit sadness. The lyrical clips and two of the instrumental clips were taken from songs in the native language of the participants. In these two instrumental clips, the lyrics were removed, such that only the instrumental music would play. The other six instrumental clips were classical pieces. Music clips were selected on the basis of a pilot test in which a separate group of participants (*N* = 60) reported their emotional reactions to 40 (19 lyrical and 21 instrumental) music clips. Participants rated their experienced sadness, gloom, and happiness. In addition, participants rated how pleasant (vs. unpleasant) they felt, and how energetic they felt after listening to each clip. Participants also rated the familiarity of the music clips. Sixteen music clips (eight lyrical and 8 instrumental) were selected as unfamiliar, such that the lyrical and instrumental clips did not significantly differ in sadness (*p* = .62), valence (*p* = .31), arousal (*p* = .56), or happiness (*p* = .37). The final list of selected clips and their descriptive statistics can be found in the online supplemental materials.

#### Procedure

**Fig. 2:**
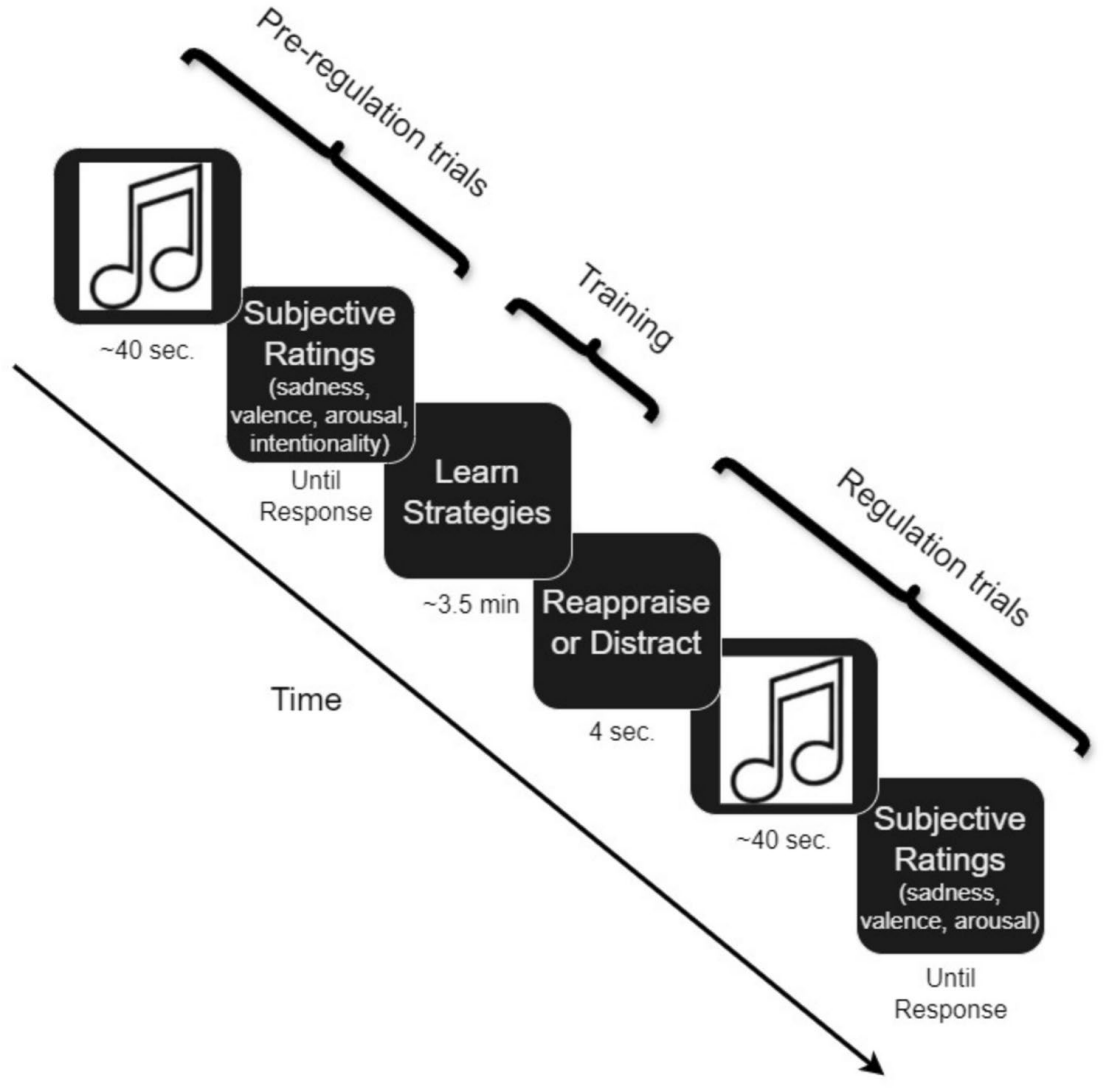
Visual Representation of the Procedure in Study 2

Figure 2 offers a visual representation of the key stages of the procedure, including the pre-regulation trials and regulation trials. Participants completed the study online. They first rated their current feelings. Participants then listened to 16 sad music clips, as well as two additional happy clips to decrease the potential for habituation. They listened to the clips in a random order and rated their affective reactions to each clip. As a manipulation check of intentionality, upon listening to each clip, participants were asked if they felt sad about something in particular (yes/no). Participants then learned how to use reappraisal and distraction. Following Shafir et al. (2015), the reappraisal instructions involved focusing on the music clips but reinterpreting their meaning, for example, by saying to oneself that what is happening will be resolved soon. Participants then practiced on emotional, but non-musical stimuli (i.e., imagining a breakup with a significant other, waking up on the wrong side of the bed and feeling cranky all day). The distraction instructions involved shifting attention away from the music clips, for example, by thinking about daily activities, familiar streets, or geometric shapes. Participants spent an average of 3.53 min (*SD* = 1.32 min) learning and practicing the strategies. Next, participants listened to the 18 music clips again in a random order. Before each clip, they were prompted to try to decrease the intensity of their emotional reactions using either distraction or reappraisal (which were presented at random). After each clip, participants rated how they felt during each clip using the same terms they rated at baseline. They also rated how difficult it was for them to implement the target strategy. As a manipulation check for strategy use, for seven of the clips, participants described what they thought about while regulating. Specifically, participants were asked, “What did you say to yourself to decrease your emotional reactions while listening to the music clip?” As pre-registered, participants who correctly described using the strategy they were instructed to implement at least four of the seven times, regardless of difficulty and success, were considered to have passed the manipulation check. Finally, participants provided demographic information, were debriefed, and thanked for their time.

## Results

### Manipulation Check

First, to confirm our piloting results and ensure that the emotion-inducing (i.e., lyrical) and mood-inducing (i.e., instrumental) clips elicited similar levels of sadness, we ran a mixed-effects model. We entered affect type (emotion vs. mood) as a Level 1 predictor and pre-regulation sadness ratings as the outcome. We included a random intercept for participant and a random intercept for stimuli. As expected, the emotion-inducing clips (*M* = 3.84, *SD* = 2.08) and the mood-inducing clips (*M* = 3.37, *SD* = 2.02) did not differ in the intensity of the sadness induced, *B* = 0.24, *SE* = 0.14, *p* = *.0*85, 95% CI [−0.03, 0.50].

Second, to ensure there was a difference in intentionality between the emotion-inducing clips and mood-inducing clips, we ran a logistic linear regression (after the random-effects multilevel model did not converge). We entered affect type as the predictor and intentionality as the outcome. As expected, participants reported feeling sad about something in particular when listening to the emotion-inducing clips more than the mood-inducing clips (28.3% vs. 19.8% of the time, respectively), *OR* = 1.59, *SE* = 0.13, *p* < *.0*01, 95% CI [1.36, 1.87], indicating that the manipulation of intentionality was successful.

### Did Affect Type Moderate the Effectiveness of Reappraisal (vs. Distraction)?

#### Changes in Sadness

**Fig. 3:**
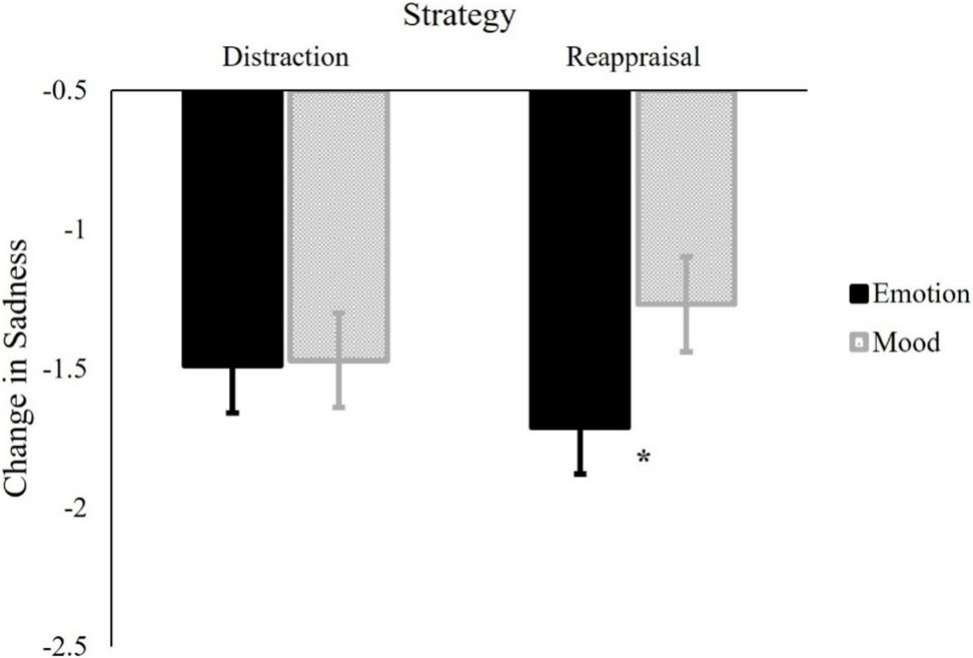
Effectiveness of Cognitive Reappraisal vs. Distraction on Regulating Sad Emotion vs. Mood. *Note.* Changes in sadness were calculated as post-regulation sadness minus pre-regulation sadness. Thus, a more negative score indicates greater success in decreasing sadness. Error bars represent standard error. * indicates *p* < 0.05

Similar to Troy et al. (2017), we operationalized emotion regulation success by subtracting affective ratings after regulation from ratings to the same clip at baseline. More negative difference scores reflect greater decreases in sadness, and hence, more successful regulation. To test our main hypothesis, we ran a mixed-effects model. We entered regulation strategy (reappraisal/distraction), affect type (emotion/mood), and their interaction as predictors, and change in sadness as the outcome. As pre-registered, we included a random intercept for participant, a random intercept for stimuli, and a by-subject random slope for regulation strategy. Similar to Study 1, our discussion here focuses on the interaction and comparison of simple effects. All effects are reported in Table 2. As predicted, we found a significant interaction between strategy and affect type, *B* = −0.10, *SE* = 0.05, *p* = .032, 95% CI [−0.20, −0.01]. To further examine the interaction, we conducted planned pairwise comparisons using estimated marginal means, using the emmeans package in R (Lenth, 2017). First, all conditions had significant decreases in sadness, *t*s < −7.32, *p*s < 0.001. As shown in Fig. 3, planned contrasts revealed that reappraisal was more successful in reducing sadness as an emotion (i.e., elicited by the lyrical music; *M* = −1.71, *SE* = 0.17) than sadness as a mood (i.e., elicited by the instrumental music; *M* = −1.27, *SE* = 0.17), *t*(36) = −2.40, *p* = .022, Cohen’s *d* = 0.29. In contrast, when using distraction, there was no significant differences between the decrease in sadness as an emotion (*M* = −1.49, *SE* = 0.17) and sadness as a mood (*M* = −1.47, *SE* = 0.17), *t*(33) = 0.15, *p* = .885. These results indicate that reappraisal (but not distraction) was more successful in reducing sadness as an emotion than sadness as a mood. Furthermore, there were no significant differences between reappraisal and distraction in changing sadness as a mood, *t*(177) = 1.29, *p* = .200, or sadness as an emotion, *t*(183) = −1.42, *p* = .159.

**Table 2:** Results of Models Predicting Change in Sadness, Valence, and Arousal

Model 1 (Sadness)					
*Predictors*	*Estimates*	*SE*	*CI*	*p*	*R*^*2*^
(Intercept)	−1.49	0.13	−1.74 – −1.23	< .001	—
Strategy	−0.01	0.06	−0.12 – 0.11	.927	0.000
Affect type	−0.12	0.07	−0.26 – 0.03	.111	0.005
Strategy * Affect type	**−0.10**	**0.05**	**−0.20 – −0.01**	**.032**	**0.004**
**Model 2 (Valence)**					
(Intercept)	0.39	0.09	0.22–0.57	< .001	—
Strategy	0.08	0.06	−0.04 – 0.19	.181	0.002
Affect type	0.12	0.05	0.02–0.21	.013	0.006
Strategy * Affect type	**0.16**	**0.05**	**0.07–0.25**	**.001**	**0.011**
**Model 3 (Arousal)**					
(Intercept)	0.32	0.10	0.13–0.51	.001	—
Strategy	−0.02	0.05	−0.13 – 0.09	.722	0.002
Affect type	0.03	0.06	−0.08 – 0.15	.539	0.000
Strategy * Affect type	**0.08**	**0.05**	**−0.02 – 0.18**	**.113**	**0.002**

Affect type was effect coded (“mood” = −1; “emotion” = 1) and Strategy was effect coded (“distraction” = −1; “reappraisal” = 1). Effects of interest (the interactions) are bolded

#### Changes in Valence

To test whether the effect replicated when assessing valence, we repeated the analysis for changes in sadness, entering changes in valence as the outcome. Larger difference scores imply greater increases in valence (more positive feelings), and hence, more successful regulation. As predicted, we found a significant interaction, *B* = 0.16, *SE* = 0.05, *p* = .001, 95% CI [0.07, 0.25]. Planned contrasts revealed that reappraisal was more successful in increasing valence elicited by the emotion-inducing music (*M* = 0.75, *SE* = 0.13) than valence due to the mood-inducing music (*M* = 0.19, *SE* = 0.13), *t*(62) = 4.00, *p* < .001, Cohen’s *d* = 0.39. As expected, when using distraction, there were no significant differences in changes in valence when regulating emotions (*M* = 0.28, *SE* = 0.12) and moods (*M* = 0.36, *SE* = 0.12), *t*(55) = 0.59, *p* = .559. These results indicate that reappraisal (and not distraction) was more successful in making people feel better when regulating emotions than moods (see Table 2, for the full results).

#### Change in Arousal

We replicated the above analyses with arousal ratings as the dependent variable. None of the effects were significant (see Table 2).

## Discussion

Study 2 provides causal support for our hypotheses Reappraisal and distraction were both effective in decreasing sadness, but the success of reappraisal (but not distraction) was moderated by whether sadness was an emotion or a mood.

## General Discussion

Our studies suggest that cognitive reappraisal may be more successful in regulating emotions than moods. Emotions, unlike moods, are about something in particular, and this key feature is precisely what cognitive reappraisal targets. This finding is consistent with past theoretical claims (Manstead & Fischer, 2000; Rottenberg & Gross, 2007), providing empirical evidence to support them in daily life and a controlled experiment.

### Theoretical and Applied Implications

The results inform our understanding of cognitive reappraisal and how or when it should be implemented. Using cognitive reappraisal is not always beneficial (Ford & Troy, 2019). We suggest that one factor that may influence the efficacy of reappraisal is the type of affect targeted for regulation. Our findings might also explain why cognitive reappraisal is not always associated with decreased negative feelings in daily life (e.g., Brans et al., 2013; Brockman et al., 2017). Given that both emotions and moods are common in daily life (Asselbergs et al., 2016; Trampe et al., 2015), it is possible that in such EMA studies, individuals were using cognitive reappraisal to regulate moods rather than emotions. If using cognitive reappraisal is less effective for regulating moods than emotions, it may be more beneficial to use it for emotion regulation, whereas other, more effective strategies could be identified to facilitate the regulation of moods.

Our results also carry broader implications for understanding the regulation of affect. This investigation may be the first to empirically test whether and how the regulation of emotions and moods might differ. Our findings provide proof of concept, that emotion regulation and mood regulation may be distinct, and that such a distinction may be important for predicting regulation success. If strategies effective for regulating emotions are not necessarily effective for regulating moods, identifying such differences could have important implications for promoting psychological health.

Our findings may also carry implications for clinical psychology, given that mood states are linked to psychopathology (Joormann & Siemer, 2014). Designing effective interventions to treat mood disorders, for instance, is critical for mental health. Our findings suggest that cognitive reappraisal is less likely to be effective when targeting mood states. Individuals with higher depressive symptoms may have lower intentionality in their affective experiences, which may limit the potential efficacy of cognitive reappraisal (Giotakos, 2023). Using cognitive reappraisal may be more effective in treating depression when it is targeted at emotional experiences, whereas other strategies may be more effective when targeting mood states.

### Limitations and Future Directions

Our studies had limitations. First, they relied on self-report measures. Self-report assessments provide information about subjective internal experiences, which is hard to obtain otherwise (Paulhus & Vazire, 2007). Nonetheless, self-report can be subjective and prone to bias. Therefore, future research can also employ physiological measures, such as skin conductance, as a proxy of emotional intensity (e.g., Kreibig et al., 2007; Wang et al., 2025), to assess regulatory success.

Second, in Study 2, although significantly different across conditions, intentionality ratings were relatively low. Future studies could use stronger manipulations of intentionality. Third, in Study 2, unlike sadness and valence, there was no effect on the regulation of arousal. In general, the effect of reappraisal on arousal has been found to be smaller than its effect on valence (e.g., Wang et al., 2024). It is, therefore, possible that the present research was underpowered to detect potential changes in arousal. Future research could try to replicate the present findings with a larger sample.

Lastly, although our findings on reappraisal were generally consistent across the two studies, these samples included predominantly young adults. Moreover, although Study 1 involved both healthy and depressed individuals, Study 2 only involved a group of healthy individuals. Given the clinical implications of mood regulation, it would be beneficial to test whether these findings could be generalized to more diverse and different samples.

## Conclusions

We demonstrate that cognitive reappraisal, which targets what an affective reaction is about, may be more useful in regulating emotions than moods (i.e., affective states that are higher vs. lower in intentionality, respectively). Our research might clarify when cognitive reappraisal is likely to be more (vs. less) effective. Our research also demonstrates that there may be differences between emotion regulation and mood regulation that should be further explored.

## Supplementary Information

Below is the link to the electronic supplementary material.

Supplementary file1 (DOCX 32 KB)

## Footnotes

1 Intentionality is distinct from cause, which is another factor that may distinguish emotion from mood. Emotions are often caused by what they are about, whereas this is not true for moods. For instance, people may feel the emotion of sadness because they failed a task, and failing the task is what they are sad about. In contrast, people may be in a sad mood because of bad weather, but they do not necessarily feel sad about the weather (Davidson, 1994). Intentionality is also distinct from emotional awareness (Boden & Thompson, 2015), which refers to the extent to which people attend to, label, and understand their affective experiences. People can attend to their feelings, label and understand them, even when their feelings lack intentionality (e.g., a person may attend to their feelings, label them as irritability, and understand they might have been induced by hormonal changes, and still not feel irritated about anything in particular).

2 As mentioned in the preregistration, the sample in the larger project involved both nondepressed and depressed individuals. In the preregistration, we initially planned to examine our research question specifically among nondepressed individuals, as depressed and nondepressed individuals might differ in how they regulate emotions in daily life (Hu et al., 2025). However, results of the present research question were not moderated by depression, therefore, we conducted the analysis on the entire sample, which was larger. Results of the nondepressed sample only are reported in the Supplemental Materials.

3 Due to within-day lagging, of the 8,206 observations, 2,575 (31.38%) included missing data for sadness at *t*-1, either because it was the first survey of the day or because participants did not complete that item. These observations were excluded, resulting in 5,631 observations for assessing prospective associations.

4 We thank an anonymous reviewer for raising this possibility.

## Additional Information

### Data Availability

All study materials, data, and R codes are available at the Open Science Framework: https://osf.io/srg93/?view_only=638758d720804c5694468378519e07b5.
