# Supplementary material for: Cognitive Reappraisal is More Effective for Regulating Emotions than Moods
Source: Affect Sci. 2025 Jun 6;6(3):477–88. doi: 10.1007/s42761-025-00310-3 (PMC12579643; doi:10.1007/s42761-025-00310-3)
Supplement: Supplementary file 1 — Supplementary file1 (DOCX 32 KB) [file 42761_2025_310_MOESM1_ESM.docx]

**Cognitive Reappraisal is More Effective for Regulating Emotions than Moods**

**Supplemental Materials**

**Table of Contents**

**Table S1. *Results of Model Predicting Prospective Changes in Sadness from Reappraisal, Affect Type, and War (only Non-Depressed Sample).*...................................................................2**

**Table S2. *Results of Models Predicting Sadness from either Distraction or Emotional Intensity, Affect Type, and War*......................................................................................................3**

**Table S3. *List of Selected Clips Used in Study 2 and their Descriptive Statistics (from the Pilot Study)*...............................................................................................................................................5**

**Table S1.** Results of Model Predicting Prospective Changes in Sadness from Reappraisal, Affect Type, and War Onset (only Non-Depressed Sample)

| *Predictors* | *Estimates* | *SE* | *CI* | *p* | *R^2^* |
| --- | --- | --- | --- | --- | --- |
| (Intercept) | 1.87 | 0.08 | 1.72 – 2.02 | <.001 | — |
| Reappraisal intensity (t-1) | -0.02 | 0.02 | -0.05 – 0.02 | .387 | .000 |
| Affect type (t-1) | 0.17 | 0.04 | 0.09 – 0.25 | <.001 | .010 |
| War onset | -0.05 | 0.08 | -0.20 – 0.10 | .539 | .001 |
| Sadness (*t*-1) | 0.12 | 0.04 | 0.04 – 0.20 | .003 | .012 |
| Reappraisal intensity (t-1)  × Affect type (t-1) | -0.01 | 0.02 | -0.05 – 0.02 | .527 | .000 |
| Reappraisal intensity (t-1) × War onset | 0.04 | 0.02 | 0.00 – 0.08 | .026 | .002 |
| Affect type (t-1) × War onset | -0.09 | 0.04 | -0.16 – -0.02 | .016 | .004 |
| **Reappraisal intensity (t-1)**  **× Affect type (t-1)**  **× War onset** | **0.04** | **0.02** | **0.01 – 0.08** | **.022** | **.002** |

***Note.*** Affect Type was effect coded (“mood” = -1; “emotion” = 1) and War onset was effect coded (pre-onset of war = -1; post-onset of war = 1). Effect of interest (the three-way interaction) is bolded.

**Table S2.** Results of Models Predicting Prospective Changes in Sadness from either Distraction or Emotional Intensity, Affect Type, and War in Study 1

| **Model 1 (Distraction)** | | | | | |  |
| --- | --- | --- | --- | --- | --- | --- |
| *Predictors* | *Estimates* | *SE* | *CI* | *p* | *R^2^* |  |
| (Intercept) | 2.63 | 0.07 | 2.48 – 2.77 | <.001 | — |  |
| Distraction intensity (t-1) | -0.00 | 0.01 | -0.02 – 0.02 | .932 | .000 |  |
| Affect type (t-1) | 0.06 | 0.03 | -0.00 – 0.11 | .068 | .001 |  |
| War onset | 0.01 | 0.07 | -0.13 – 0.16 | .874 | .000 |  |
| Sadness (*t*-1) | 0.25 | 0.02 | 0.20 – 0.29 | <.001 | .049 |  |
| Depressive status | 0.83 | 0.07 | 0.68 – 0.97 | <.001 | .233 |  |
| Distraction intensity (t-1)  × Affect type (t-1) | 0.00 | 0.01 | -0.02 – 0.02 | .864 | .000 |  |
| Distraction intensity (t-1) × War onset | 0.02 | 0.01 | -0.01 – 0.04 | .154 | .000 |  |
| Affect type (t-1) × War onset | -0.04 | 0.03 | -0.09 – 0.02 | .177 | .001 |  |
| **Distraction intensity (t-1) × Affect Type (*t*-1)** | **-0.00** | **0.01** | **-0.02 – 0.02** | **.907** | **.000** |  |
| **Model 2 (Emotional Intensity)** | | | | | | |
| (Intercept) | 2.60 | 0.07 | 2.46 – 2.75 | <.001 | — |  |
| Reappraisal intensity (*t*-1) | -0.02 | 0.02 | -0.05 – 0.01 | .254 | .000 |  |
| Sadness (*t*-1) | 0.27 | 0.02 | 0.23 – 0.31 | <.001 | .069 |  |
| War onset | 0.03 | 0.07 | -0.12 – 0.17 | .718 | .002 |  |
| Depressive status | 0.84 | 0.07 | 0.69 – 0.98 | <.001 | .241 |  |
| Reappraisal intensity (*t*-1) × Sadness (*t*-1) | -0.01 | 0.01 | -0.03 – 0.00 | .059 | .001 |  |
| Reappraisal intensity (*t*-1) × War onset (*t*-1) | 0.01 | 0.02 | -0.02 – 0.04 | .434 | .000 |  |
| Sadness (*t*-1) × War onset (*t*-1) | -0.02 | 0.02 | -0.06 – 0.03 | .457 | .000 |  |
| **Reappraisal intensity (*t*-1) × Sadness (*t*-1)**  **× War onset (*t*-1)** | **0.01** | **0.01** | **-0.00 – 0.03** | **.111** | **.001** |  |

***Note.*** Affect Type was effect coded (“mood” = -1; “emotion” = 1), depressive status was effect coded (“Control” = -1; “Depression” = 1), and War onset was effect coded (pre-onset of war = -1; post-onset of war = 1). Effect of interest (the three-way interaction) is bolded.

**Table S3.** List of Selected Clips Used in Study 2 and their Descriptive Statistics (from the Pilot)

| Title | Artist | Start-End times | Music Type | Sadness | Happiness | Arousal | Valence | Familiarity |
| --- | --- | --- | --- | --- | --- | --- | --- | --- |
| Vilja-Lied | Franz Lehár | 2:05-2:30 | instrumental | 3.55 | 3.17 | 4.27 | 4.67 | 1.8 |
| Adagio for Strings - Opus 11 | Samuel Barber | 3:56-4:35 | instrumental | 3.83 | 3.12 | 4.03 | 4.53 | 2.12 |
| Swan of Tuonela | Jean Sibelius | 2:20-2:55 | instrumental | 4.16 | 2.35 | 4.06 | 4.19 | 1.48 |
| Adagio for Strings - Opus 11 | Samuel Barber | 0:00-0:45 | instrumental | 4.43 | 2.2 | 3.83 | 4 | 1.83 |
| Symphony No. 5 | Gustav Mahler | 1:00-1:52 | instrumental | 4.93 | 2.23 | 3.97 | 3.7 | 1.97 |
| Mixture of sad classical music from the above list | N/A | 1:00-1:45 | instrumental | 4.76 | 2.28 | 3.72 | 3.59 | 2.45 |
| Pa'am Rishona | Daniel Kitchels | 0:24-1:04 | instrumental* | 3.53 | 2.84 | 3.74 | 4.58 | 1.55 |
| Baruch Haba | Gabriel Balachsan | 0:18-0:32 | instrumental* | 3.66 | 2.72 | 4.07 | 4.28 | 1.24 |
| Mitgagaya | Oshik Levy | 0:54-1:36 | lyrical | 3.27 | 2.43 | 4.43 | 4.8 | 2 |
| Hutim Shel Chesed | Gabriel Balachsan | 0:46-1:35 | lyrical | 3.97 | 1.41 | 2.47 | 2.41 | 1.53 |
| Zehu Zeh | Alon Elder | 1:07-1:51 | lyrical | 4.09 | 2.07 | 4.1 | 3.52 | 2.69 |
| Habechi | Oshik Levy | 1:10-1:49 | lyrical | 4.1 | 3.06 | 4.87 | 4.39 | 2.03 |
| Hi Borachat | Daniel Rubin | 0:00-0:42 | lyrical | 4.16 | 2.24 | 4.62 | 4 | 1.62 |
| Im Kvar Levad | Micha Shitrit | 1:36-2:10 | lyrical | 4.22 | 2.4 | 3.57 | 3.9 | 2.97 |
| L'lo Milim | Shalom Hanoch | 0:09-0:54 | lyrical | 4.73 | 2.41 | 3.69 | 3.81 | 1.88 |
| Yom Echad | Oshik Levy | 0:10-0:44 | lyrical | 4.86 | 2.94 | 3.22 | 4.12 | 1.69 |

***Note.*** * indicates that the song was originally lyrical, but that the lyrics were removed for it to be only instrumental. All measures were rated on a 1-9 scale.
